# Exploring the Early Molecular Pathogenesis of Osteoarthritis Using Differential Network Analysis of Human Synovial Fluid

**DOI:** 10.1016/j.mcpro.2024.100785

**Published:** 2024-05-14

**Authors:** Martin Rydén, Amanda Sjögren, Patrik Önnerfjord, Aleksandra Turkiewicz, Jon Tjörnstrand, Martin Englund, Neserin Ali

**Affiliations:** 1 Clinical Epidemiology Unit, Department of Clinical Sciences Lund, Orthopedics, Faculty of Medicine, Lund University, Lund, Sweden; 2 Department of Clinical Sciences Lund, Rheumatology, Rheumatology and Molecular Skeletal Biology, Faculty of Medicine, Lund University, Lund, Sweden; 3 Department of Orthopaedics, Skåne University Hospital, Lund, Sweden

**Keywords:** osteoarthritis, synovial fluid, proteomics, SOMAscan assay, Gaussian graphical models, protein networks

## Abstract

The molecular mechanisms that drive the onset and development of osteoarthritis (OA) remain largely unknown. In this exploratory study, we used a proteomic platform (SOMAscan assay) to measure the relative abundance of more than 6000 proteins in synovial fluid (SF) from knees of human donors with healthy or mildly degenerated tissues, and knees with late-stage OA from patients undergoing knee replacement surgery. Using a linear mixed effects model, we estimated the differential abundance of 6251 proteins between the three groups. We found 583 proteins upregulated in the late-stage OA, including MMP1, collagenase 3 and interleukin-6. Further, we selected 760 proteins (800 aptamers) based on absolute fold changes between the healthy and mild degeneration groups. To those, we applied Gaussian Graphical Models (GGMs) to analyze the conditional dependence of proteins and to identify key proteins and subnetworks involved in early OA pathogenesis. After regularization and stability selection, we identified 102 proteins involved in GGM networks. Notably, network complexity was lost in the protein graph for mild degeneration when compared to controls, suggesting a disruption in the regular protein interplay. Furthermore, among our main findings were several downregulated (in mild degeneration *versus* healthy) proteins with unique interactions in the healthy group, one of which, SLCO5A1, has not previously been associated with OA. Our results suggest that this protein is important for healthy joint function. Further, our data suggests that SF proteomics, combined with GGMs, can reveal novel insights into the molecular pathogenesis and identification of biomarker candidates for early-stage OA.

## Highlights

• High-throughput SOMAscan assay proteomics of human synovial fluid (SF).
• SF from knees with mildly degenerated tissue, representing early osteoarthritis (OA).
• Explorative and data-driven workflow to find novel biomarkers of early OA.
• Analysis of protein-protein dependencies using Gaussian Graphical Models (GGMs).
• Loss of network complexity in the mild degeneration group when compared to controls.

## Graphical Abstract

**Figure:**
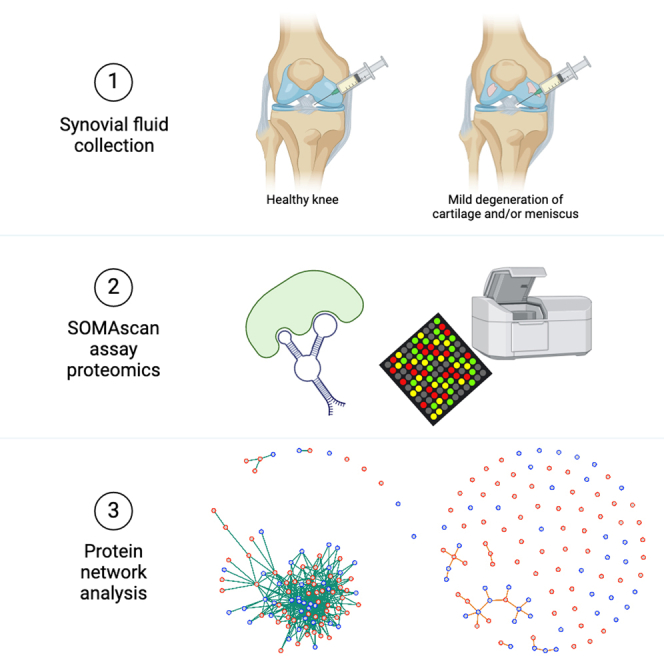

## In Brief

This study presents SOMAscan assay proteomics data from human SF. SF was obtained from three groups, including knee joints with signs of mild degeneration. This group may represent early-stage knee OA, an underrepresented group in OA biomarker studies. Apart from conventional differential abundant analysis, we conducted network analysis of protein-protein dependencies using GGMs. Notably, network complexity was found to be lost in the graph representing early-stage OA, as compared to controls, suggesting a disruption in the regular protein interplay.

Osteoarthritis (OA) is a degenerative joint disease that mostly affects the knees, hips, and hands, leading to symptoms such as pain, stiffness, and reduced joint function. The knee is the joint most frequently affected by OA. In the knee, the disease affects the whole joint and is characterized by irreversible loss of articular cartilage, synovial inflammation, remodeling of the subchondral bone, and degeneration of the menisci. The most well-established risk factors associated with knee OA are aging, high body mass index, joint injury, genetics, and female sex (1). OA spans globally and in 2020, around 654 million individuals aged 40 years or older, were reported to be affected by knee OA alone (2). Currently, there are no disease-modifying treatments available, and thus, understanding the underlying molecular mechanisms involved in the development and progression of OA is crucial for developing new effective therapies (3).

Early-stage OA is characterized by subchondral bone loss, breakdown of aggrecan, and synovial inflammation. These changes in the joint tissues may interact together, and with systemic factors such as aging, obesity, and genetics, to drive OA pathogenesis (4). As most previous studies focus on established or late-stage knee OA, there is still a lack of knowledge about early disease mechanisms (5). While a number of different omics analyses have been carried out separately to reveal the underlying mechanisms of OA (6, 7, 8), no consensus has yet been reached pinpointing the molecular events that drive OA initiation and progression (9, 10, 11). In our previous proteomics studies, we have detected altered interplay between proteins in the synovial fluid (SF) of the knee, in a disease-stage-dependent manner (12, 13). Our results point to the need to approach the proteome of the joint as a network of interacting proteins, to further unravel the complex nature of protein interactions involved in the disruption of joint homeostasis in OA. Using large-scale protein data, such interplay between proteins can be analyzed and visualized with the use of graph theory. In short, each protein is represented as a node, and connections between proteins are represented with edges (14). A specific type of tool in graph theory is Gaussian Graphical Models (GGMs), which enable direct dependency between variables to be visualized (15). Thus, using GGMs, protein data is represented as an interconnected network with conditional dependencies between proteins, which enables the identification of important subnetworks or protein hubs potentially involved in the disease.

SF is located between knee joints and is therefore of interest to study with regard to secreted proteins from tissues affected by OA (16). However, studying the SF presents challenges due to its complexity and high variability in protein intensities. The SF proteome is dominated by highly abundant proteins, which can mask and bias against the detection of low abundant proteins (17). Moreover, previous proteomics studies on SF changes in knee OA have only identified a fraction of the whole proteome present in SF. In this exploratory study, our goal was to extend the existing mapping of the SF proteome using the SOMAscan assay, a proteomic platform that utilizes aptamers as reagents to detect and quantify, on a relative scale, up to 7000 proteins in a biological sample (18). Ultimately, our aim was to model protein networks in human SF from healthy knee joints or with mild cartilage and/or meniscus degeneration and apply GGMs to analyze data from the SOMAscan assay (19).

## Experimental Procedures

### Experimental Design and Statistical Rationale

SF from human knees was obtained from the MENIX biobank at Skåne University Hospital, Lund, Sweden. We used samples from deceased donors and from patients who have undergone total knee replacement. All knees were from independent donors and patients. All deceased donor samples from MENIX were obtained within 48 h post-mortem, and the specimens were frozen at −80 °C within 2 h of extraction. SF was centrifuged for 10 min at 1800 rcf before freezing. We selected SF from two groups of deceased donors. Group 1: donors with visual signs of mild degeneration in cartilage and/or menisci (mean [SD] age 71.4 years [7.0], n = 13; 7 males and 6 females), hereafter referred to as the mild degeneration group. Group 2: deceased donors, without visual evidence of tibiofemoral OA or known clinical knee OA (mean [SD] age 70.7 [11.4], n = 12; 7 males and 5 females), hereafter referred to as healthy controls. For selection into these groups, cartilage and menisci were macroscopically graded by two independent observers, and consensus was reached for discrepancies. For the controls, cartilage surfaces and menisci were both required to be smooth and intact. Representative images of articular cartilage from the healthy and the mild degeneration group can be seen in Supplemental Fig. S1. Furthermore, a third group of samples from patient donors undergoing total knee replacement due to medial late-stage OA (mean [SD] age 71.1 years [5.6], n = 14; 8 males and 6 females) was also selected, hereafter referred to as the late stage OA group. Informed consent was obtained before the collection of patient tissues. The sample collection and analysis have been proven by the ethical review committee of Lund University (Durs: 2015/39;2016/865; 2019/3239) and carried out in accordance with relevant guidelines and regulations by the Declaration of Helsinki principles. The sample size was based on sample availability, and we balanced the groups with respect to age and sex.

### SOMAscan Assay Proteomics

For each sample in the study, 130 μl of SF treated with 10 μl hyaluronidase (Sigma, 1 μg/μl at 37 °C for 3 h) were shipped to Somalogic in the US for SOMAscan analysis. 55 μl of each sample was utilized in the analysis. Each sample was divided into three dilution sets: 20%, 0.005%, and 0.5%, to achieve a wide dynamic range, and data was collected using the SOMAscan Assay v4.1 (18). In short, fluorophore-tagged aptamers (7288 unique aptamers mapping to 6383 unique UniProt IDs) selectively bind to their target proteins in the sample, while a polyanionic competitor added to the buffer forces nonspecific aptamer-protein interactions to disassociate. Finally, the relative abundances of the target proteins are indirectly measured using the fluorescent tags on their specifically bound aptamers *via* hybridization sequencing. Several types of normalization steps are carried out: (1) Hybridization Control Normalization: 12 control sequences are added before hybridization to control for the variability in the readout, (2) Intraplate Median Signal Normalization: in each plate, calibrator samples (n = 5) and buffer samples (n = 3) are assigned a scale factor to account for systematic variability, (3) Plate Scaling and Calibration: calibrators (pooled plasma samples) are used to account for variation between plates and (4) Median Signal Normalization to a Reference: a median normalization step can be applied to each sample to achieve fully normalized datasets (18). To evaluate repeatability, six samples were pipetted again and treated in the same way as described above. Repeatability coefficients were calculated for all aptamers with quantification values above the limit of detection in all 12 samples. Calculations were done on normalized data on the log2 scale.

### Statistical Analysis

Differential abundance analysis of SOMAscan assay data was done at the aptamer level on log2 transformed values (20), with the main goal of finding suitable aptamers to include in the GGMs. We excluded aptamers that (1) were not mapped to any UniProt-ID (34 aptamers), (2) had target proteins mapped to non-human organisms (261 aptamers) and (3) had a quantification signal lower than the limit of detection in any sample (213 aptamers). The limit of detection was calculated based on log2 transformed values, using a threshold value of the buffer’s mean plus five times its standard deviation. This resulted in 2.9% of the aptamers being filtered out and a total of 7088 aptamers (6251 proteins) were left for further analysis. To have feasible runtimes but retain the advantage of shrinkage of estimates and standard errors (and thus also false positive rates), linear mixed effect models were iteratively conducted by including 50 randomly selected aptamers at a time. Study groups (mild degeneration, late-stage OA, and healthy controls), sex, and age were used as fixed effects, while the specific study subject was treated as a random effect to take into account the clustering of aptamers within a given donor (21). Contrasts between groups (mild degeneration *versus* control and late-stage OA *versus* control) were specified using the emmeans package and reported with 95% confidence intervals (95% CIs) based on restricted maximum likelihood estimates using the Kenward-Rogers method for estimation of degrees of freedom. Although our main aim was to derive the GGMs in healthy and mild degeneration groups, we included the late-stage OA group in the differential abundance analysis for two reasons: (1) to include more data in the model (for better estimation of the confounding effect of age and sex, as well as variability in the aptamers) and (2) to provide estimates for differences in aptamer levels between late stage OA and healthy controls, for completeness.

### Gaussian Graphical Models

A GGM is a network (graph) model that uses the partial correlation coefficient to measure the conditional dependence between variables, given all other variables. The nodes of the graph correspond to the variables (proteins), and the absence of an edge between two nodes implies that they are conditionally independent given the other variables, *i.e.*, two proteins are conditionally independent given a set of other variables if the distribution of the two variables does not change when conditioned on the other variables. GGMs are sparse, meaning that only pairs of variables that are directly related have an edge between them. A GGM can filter out the effects of confounding or mediating variables and may reveal the underlying causal structure of the data (22). In the study of biological systems, GGMs are used as exploratory research tools that can be used to infer interesting relations between proteins including their interactions and functional clusters (15, 23).

The jewel method (24), implemented as an R-package (19), is a technique for estimating GGMs from high-dimensional datasets that share some dependency structure. The method uses a node-wise regression approach with a group lasso penalty to enforce the symmetry and joint learning of the graphs. A feature of the Jewel 2.0 method is the stability selection procedure which reduces the number of false positives in the estimated graphs using resampling and consecutive model constructions. In the end, edges that are present in 80% (default) of such models are considered as true positives and retained in the final model. The GGM implementations all rely on the assumption that the similarities and dissimilarities in the networks are of comparable sizes between all the groups (19), which made us exclude the late-stage OA in the network model and focus on the group of mild degeneration, which we find most interesting. To summarize, in this study, we used the Jewel 2.0 method to estimate the proteomic interactome of SF from healthy and mildly degenerated knee joints.

To have computationally feasible models, we selected a set of 760 proteins (corresponding to 800 aptamers in the SOMAscan assay), with the largest absolute fold changes from the linear mixed effect models, to be included in the GGMs. After the models were constructed, and to make biological interpretation of the networks possible, each node was represented by the corresponding protein. Visualization of the networks was done with the *igraph* R package (25).

**Supplemental Figure S2:**
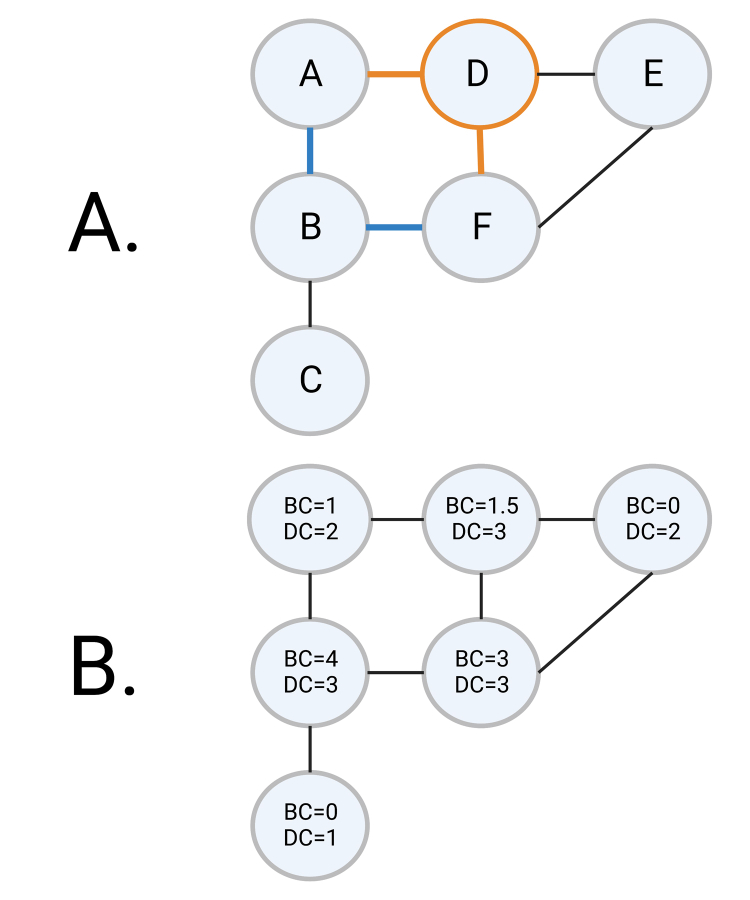
*A*, an undirected graph with nodes *A*–*F*. Each line represents an edge. A path is a collection of connected edges. The degree of node D is three, as there are three edges directly connected with node D. Accordingly, the degree of node C is one. To determine the betweenness score of node D, the shortest paths between all other pairs of nodes are considered. As an example, the shortest paths between *A* and *F* are shown with *blue* and *orange*, where the *orange* path passes through node D. The total number of shortest paths, and the number of shortest paths that passes through node D, are also calculated for each other pair of nodes in the network. The percentage of shortest paths that passes through node D is calculated for each pair of nodes and summed up to generate the betweenness score for node D. The same procedure is repeated for each node. *B*, betweenness centrality (BC) and degree centrality (DC) scores for all nodes in the graph. Note that a node can have the same degree centrality, but a different betweenness centrality score, and vice versa.

The importance of a node in a graph can be quantified by different types of centrality measures. We focused on two types for our GGMs. Namely, degree centrality and betweenness centrality (26, 27). These measures have previously been identified as important for cancer proteins as compared to essential and control genes/proteins (28). The degree centrality of a node simply represents the total number of edges directly connected to the node and the higher the score, the more central the node is. The betweenness centrality score, on the other hand, is a measure of how much a specific node influences the flow through the entire graph. In other words, it is a measure of the number of paths passing through this node (example of calculations in Supplemental Fig. S2).

#### Selection of Regularization Parameters

In the selection process for optimal regularization parameters (λ_1_ and λ_2_), we evaluated the Bayesian Information Criterion (BIC) for a range of values (0.01–0.25) for λ_1_ (while keeping a fixed λ_2_ at 0.001 value) and then for a range of values (0.0001–0.1) for λ_2_ (while keeping λ_1_ fixed at the preselected value) (29). Stability selection was not used in the evaluation of model fit. After evaluating the sparseness of the models, and the model fit using BIC, we used λ_1_ = 0.1 and λ_2_ = 0.001, to construct the final models.

#### Model Construction

Stability selection with 1000 resampled subsets was used to reduce the number of false positives. This procedure includes a fixed number of randomly selected aptamers and iteratively evaluates the network. For the final models, we included edges between aptamers that were present in 80% of the networks (default) during the stability selection procedure. In the final models, after applying stability selection with 1000 subsets, 103 aptamers remained.

#### Community Detection and Characterization

We examined the presence of communities (subnetworks) using the cluster_louvain function in R. In this function, nodes are stepwise divided into communities *via* hierarchical assignments using modularity measures (30). The function was run 20 times and the final community structure reported was the most frequent one. Communities with three or more members were labeled alphabetically.

### Classification

The functional classification of proteins was performed using the PANTHER knowledgebase (31). Here, we used the ontological term “PANTHER protein class” by calling the web services for gene information using the PANTHER API (the full script is available on GitHub). This classification system includes commonly used classes of protein functions which allowed us to provide a concise functional description of the proteins identified in this study. Due to the degenerative nature of OA, proteins active in the extracellular matrix (ECM) can be expected to secrete into the SF of the osteoarthritic joint. We therefore had a particular interest in evaluating the prevalence of ECM proteins among the selected proteins.

### Validation with Mass Spectrometry Proteomics

To validate the results from the SOMAscan assay, we collected mass spectrometry (MS) data. Because of a limited amount of synovial fluid, three samples in both mild degeneration and control groups were excluded in the acquisition of validation data. Thus, we included ten samples in the mild degeneration group (mean [SD] age 71.5 years [7.0]; 6 males and 4 females) and nine samples in the healthy group (mean [SD] age 70.7 years [11.4], 5 males and 4 females). We had enough material for MS analysis for all the samples in the late-stage OA group.

#### Sample Preparation

For MS analysis, 25 μl SF was mixed with 10 μl of MS-safe proteinase inhibitor cocktail and 10 μl hyaluronidase (1 μl/μg) and incubated at 37 °C for 3 h. The 7 most abundant proteins were depleted with the multiple affinity removal systems (MARS Hu7 spin cartridge) according to the manufacturer's protocol (Agilent Technologies). Samples were reduced with 4 mM dithiothreitol and kept at +56 °C for 30 min during shaking. Thereafter, samples were alkylated with 16 mM iodoacetamide and kept in the dark for 1 h at room temperature. 95% ethanol with 50 mM NaAc was used in a 1:9 volume to precipitate samples. Samples were left at 4 °C overnight. The day after, samples were centrifuged at 13,000 rpm for 1 h and the supernatant was removed from the pellets. Pellets were dissolved in 0.1 M ammonium bicarbonate followed by protein digestion overnight at 37 °C with sequencing grade trypsin (Promega) at a protease/protein ratio of 1:50. Next, samples were subjected to a 30 kDa filter (Pall Corporation, 96 well) and a vacuum pump (Rocker 400, VacMaster VCU) to remove large compounds from the sample and reduce the risk of clogging the MS column. Additionally, samples were desalted with C18 AssayMAP cartridges using the Bravo platform (Agilent Technologies). Thereafter, samples were evaporated and dissolved in 20 μl of 0.1% formic acid. At last, samples were further spiked with iRT peptides, for retention time normalization, before analyses with MS.

#### Data Collection

The samples were run in randomized order on an EASY-nLC 1000 (Thermo Scientific) coupled to a Thermo Scientific Q-Exactive HFX mass spectrometer using data-independent acquisition (DIA). The mobile phases A and B were 0.1% formic acid and acetonitrile with 0.1% formic acid, respectively. Peptides were loaded on an Acclaim PepMap 100 nanoViper pre-column (Thermo Scientific, C18, 3 μm particles, 75 μm i.d. 2 cm long) at 5 μl/min mobile phase A. Peptide separation was carried out on a PepMap RSLC C18 analytical column (Thermo Scientific, C18, 2 μm particles, 75 μm i.d. 25 cm long) at 45 °C and 300 nl/min using a gradient. Mobile phase B was initially kept at 5 to 7% for 5 min followed by 85 min at 7 to 20%, 20 min at 20 to 30%, 5 min at 30 to 90%, and finally, held at 90% for 5 min. To equilibrate the column after each gradient, the mobile phase B was kept at 3% for 15 min. Settings for the DIA method was as follows: duration 125 min, full scan resolution 120,000, scan range 350 to 1650 m/z, AGC target 3.0e6, maximum injection time 100 ms, Orbitrap resolution 45,000, AGC 3.0e5 with a variable isolation window 33/26/22/20 × 2/18/20/19 × 4/20/21 × 2/23 × 2/24/26/31/32/37/40/53/66/99/574 m/z (Supplemental Data S1), and normalized collision energy 27 eV, giving a cycle time of 3.3 s.

#### Spectronaut Search

The raw data were analyzed with SpectronautPulsar software (version 18.4.231017.55695, Biognosys AG, Switzerland) for protein identification and quantification using directDIA (library-free) search, and global normalization (median). We used human protein fasta files from the uniport database (20,230,913; 20,586 protein entries). In addition to default settings, cysteine carbamidomethylation was used as a fixed modification. Furthermore, acetylation (N-term), oxidation (M), and oxidation (P) were used as variable modifications. Trypsin/P was specified as a specific digestion type with a maximum of two missed cleavages. Quantification of MS2 precursor ions was done by area under the curve quantitation. The 3 most abundant proteotypic peptides (mean) and MaxLFQ were used for protein quantification. Default settings were used for MS1 and MS2 mass tolerance (40 ppm). Peptide and protein false discovery rate was 0.01.

#### Data Analysis

To extract proteins of interest for validation purposes, we looked at the overlap for the 102 proteins in the GGMs with the MS data. This resulted in a total of 31 overlapping proteins. To be kept in the analysis the following criteria had to be fulfilled: (1) a protein needed to have a proteotypic peptide, *i.e.* a peptide uniquely mapped to the FASTA file (4 proteins filtered out), (2) a protein needed to have more than one (stripped; no modifications) peptide used for identification in the data set (3 proteins filtered out), and finally (3) a protein needed to have more than 50% non-missing values (3 proteins filtered out). In the end, we analyzed 21 overlapping proteins by (1) estimating the correlation between the methods on log2 standardized data sets and (2) comparing log2 fold changes and 95%CIs by linear mixed effect model with quantification on the protein level as the outcome variable. The variables were study group, UniProt-ID, age, sex, and interactions between study group and UniProt-ID, age and UniProt-ID, and finally, sex and UniProt-ID. The subjects/patient-IDs were used as a random effect.

### SLCO5A1: Antibody Validation by Western Blotting

SLCO5A1, which was not identified with MS, was identified using an antibody. We chose to analyze the sample with the highest SLCO5A1 abundance according to the SOMAscan assay. SF was subjected to Top 14 Abundant Protein depletion spin column (ThermoFisher Scientific, A36370) to remove highly abundant proteins. After, samples were concentrated by Vivaspin 500 centrifugal concentrators (Merck, Z614041) to achieve a volume suitable for SDS-PAGE. 2 μl 0.5 M DTT and 5 μl LDS Sample Buffer (Thermo Fisher Scientific, 1981103) were added. Further, MilliQ water was added to achieve a final volume of 20 μl. Samples were incubated for 10 min at 70 °C and then subjected to a NuPAGE 4 to 12% Bis-Tris Gel in MOPS buffer (ThermoFisher Scientific, 2711057) at 200 V for 50 min. Proteins were transferred to a polyvinylidene difluoride membrane in a transfer buffer (ThermoFisher Scientific, 2461350) with 10% methanol at 25 V for 90 min. The membrane was blocked with 5% non-fat dry milk in TBS with 0.05% Tween. The primary antibody (SLCO5A1, Nordic Biosite, LS-B11930) was diluted 1:1000 and incubation was carried out overnight at 4 °C. The next day, the secondary antibody (P0217 Dako) was diluted 1:1000 and incubated for 1 h at room temperature. SuperSignal West Dura kit was used as a chemiluminescent substrate, and 10 min exposure was needed to visualize any protein bands on the membrane (Bio-Rad, ChemiDoc MP Imaging System).

## Results

### Differential Expression

The late-stage OA group had more proteins with high absolute fold change as compared to the mild degeneration group. As an example, a total of 104 proteins with an absolute fold change larger than 2 can be found in the late-stage OA group, whereas only one can be found in the mild degeneration group. In the late-stage OA group, we found differentially expressed proteins of matrix metalloproteinases (MMPs) such as: MMP1 (log2 fold change 4.31, 95% CI [3.85, 4.76]), collagenase 3 (2.21 [1.76, 2.67]) and stromelysin-1 (1.89, [1.43, 2.35]). These proteins degrade ECM components and are well-studied as OA biomarkers (32, 33, 34, 35, 36). Furthermore, among the proteins with highest absolute log2 fold change we found upregulation of inhibin beta A chain (3.58, [3.04, 4.11]), tumor necrosis factor-inducible gene 6 protein (2.60, [2.09, 3.12]) and kininogen-1 (2.60, [2.13, 3.07]) as well as downregulation of NAD(P)H dehydrogenase (quinone) 1 (−3.29, [−3.79, −2.79]), hemoglobin subunit alpha (−3.25, [−3.79, −2.71]) and aldehyde dehydrogenase, mitochondrial (−2.87, [−3.37, −2.37]) (Supplemental Data S2). We also identified some proteins that, to our knowledge, have not previously been associated with OA, such as ADH1C (−5.51, [−6.04, −4.97]) and GPD1 (−5,28, [−6.04, −4.97]). For the mild degeneration group, proteins among those with the highest absolute log2 fold change were TCN2 (−1.48, [−2.10, −0.85]), PADI4 (1.34, [0.77, 1.91]), bactericidal permeability-increasing protein (1.01, [0.56, 1.46] and insulin-like growth factor-binding protein 2 (−0.98, [−1.42, −0.54]) (Supplemental Data S2).

### Gaussian Graphical Models

**Fig. 1:**
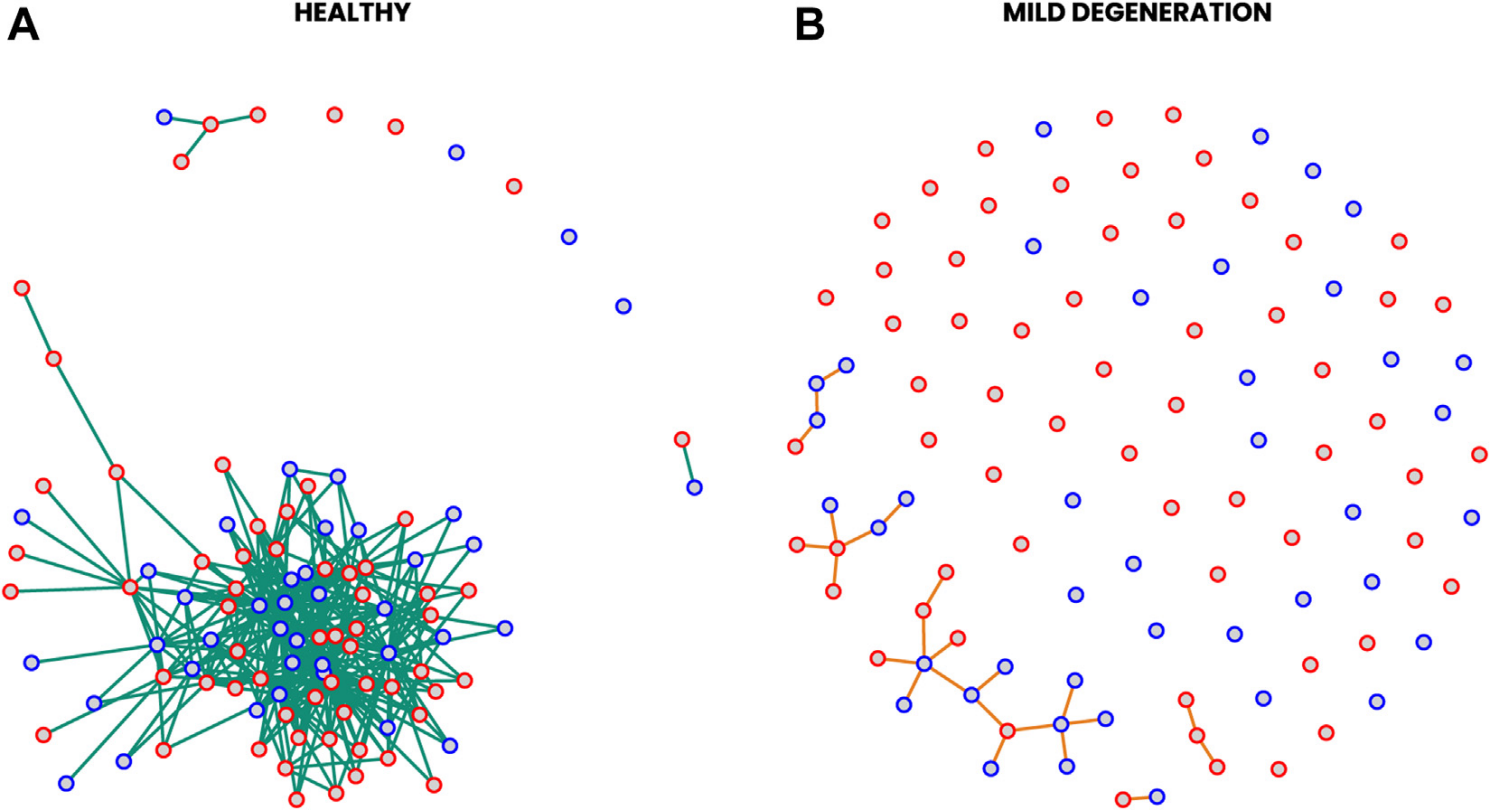
**Protein-protein interaction networks derived from Gaussian Graphical models (GGMs) for two study groups: healthy controls (Panel A) and mild degeneration (Panel B).** Only nodes with unique edges in the respective group were included. Each node in the network signifies a protein, with edges representing conditional dependencies or interactions between two proteins. Frames of nodes are colored *blue* and *red* to indicate upregulated and downregulated proteins (mild degeneration in comparison to healthy controls), respectively. *Green* edges are exclusive to healthy controls, and *orange* edges are exclusive to the mild degeneration group. The Fruchterman-Reingold algorithm was used for layout and the same protein may have different positions in the two networks. The absence of a node indicates that the corresponding protein had no detected interactions in either condition.

To construct the GGMs of the mild degeneration group and the healthy control group, we included 760 unique proteins (corresponding to 800 aptamers with the highest absolute fold changes, Supplemental Data S2). In the final models, the two networks included 102 proteins (corresponding to 103 aptamers, Supplemental Data S3). With regard to model similarities, 87 of the protein nodes share a total of 2472 edges (Supplemental Fig. S3). Model differences can easily be observed by excluding shared edges (Fig. 1). More specifically, in the network for the mild degeneration group, there are 29 proteins with 48 unique edges, whereas the control group network has a total of 752 unique edges connected to 97 protein nodes.

We also examined the presence of community structures in our data (Supplemental Fig. S4). Here, we defined a community as a subnetwork consisting of three or more connected nodes. In the analysis of GGMs with unique edges, we identified five such communities in the controls and six in the mild degeneration group.

We used centrality scores of betweenness to identify proteins of interest. First, we included all edges (Supplemental Data S3). The protein with the corresponding gene name TPM3 had the highest betweenness centrality score of 971 in the mild degeneration group (log2 fold change 0.46, 95% CI [−0.01, 0.94]; degree centrality 44). Likewise, this protein also has the highest betweenness score in the control group. To capture as much of a difference as possible between the two networks, we used a second approach to base the centrality scores solely on unique edges (Supplemental Data S4). This approach allowed us to discern the group-specific protein interplay while simultaneously reducing the complexity of the network.

The top ten proteins based on betweenness score are displayed for the healthy control group (Table 1) and mild degeneration (Table 2). A high betweenness score indicates that the protein is of importance for the flow of information through the network and can be considered as a bottleneck protein. Considering this, a few of the proteins within the mild degeneration group have notably higher betweenness centrality: HIBCH, DHX8, A1CF, and PHF3. In the healthy group, SLCO5A1 has the highest betweenness score and among the highest degree score, which suggests that this protein might be important for healthy joint function.

**Table 2:** Proteins with unique edges in the mild degeneration group’s network

Community	Entrez Gene	Classification	Degree centrality	Betweenness centrality	Log2 fold change	95% CI
F	FAM3D	antimicrobial response protein	4	9	−0.49	(−0.93, −0.05)
F	H1-10	chromatin/chromatin-binding, or -regulatory protein	2	4	0.87	(0.41, 1.32)
G	DNAJB2	chaperone	2	1	−0.51	(−1.12, 0.09)
H	HIBCH	hydrolase	5	49	0.77	(0.34, 1.20)
H	SOCS3	kinase modulator	2	12	−0.59	(−1.11, −0.07)
I	DHX8	RNA helicase	3	48	0.44	(−0.06, 0.95)
I	A1CF	RNA metabolism protein	3	44	−0.43	(−0.85, −0.01)
J	PHF3	general transcription factor	4	33	0.53	(0.09, 0.98)
K	ACBD6	*NA*	2	2	0.50	(0.00, 0.99)
K	MVK	carbohydrate kinase	2	2	0.43	(−0.05, 0.90)

The 10 proteins with highest betweenness scores are displayed. This table represents these proteins’ community, Entrez Gene, Classification (where applicable), centrality scores (degree and betweenness), Log2 fold change, and 95% confidence interval (95% CI). A positive Log2 fold change means that the protein is upregulated in the mild degeneration group as compared to healthy controls.

### Classification

Out of the 6414 proteins identified in this study, we were able to describe the function of 4670 proteins (73%) using the PANTHER knowledgebase for protein classification (Supplemental Data S5). The most common classifications among the identified proteins were transmembrane signal receptor (240 proteins), scaffold/adaptor protein (215 proteins), and ubiquitin-protein ligase (122 proteins). The ECM categories (extracellular matrix structural protein, extracellular matrix protein, extracellular matrix glycoprotein) constituted 72 proteins. Among differential expressed proteins in the mild degeneration group, examples of proteins in the ECM categories are: aggrecan core protein (ACAN), different types of collagens, fibronectin (FN1), hyaluronan and proteoglycan link protein 1 (HAPLN1) and von Willebrand factor (VWF) (Supplemental Datas S2 and S5). In proteins with unique edges (Fig. 1), only fibulin-5 (FBLN5) belonged to the ECM category.

### Repeatability

Repeatability coefficients (Supplemental Data S6) for the aptamers had a median (first and third quartile) of 13% (9% and 20%), which indicates excellent repeatability.

### Validation with Mass Spectrometry Proteomics

**Supplemental Figure S5:**
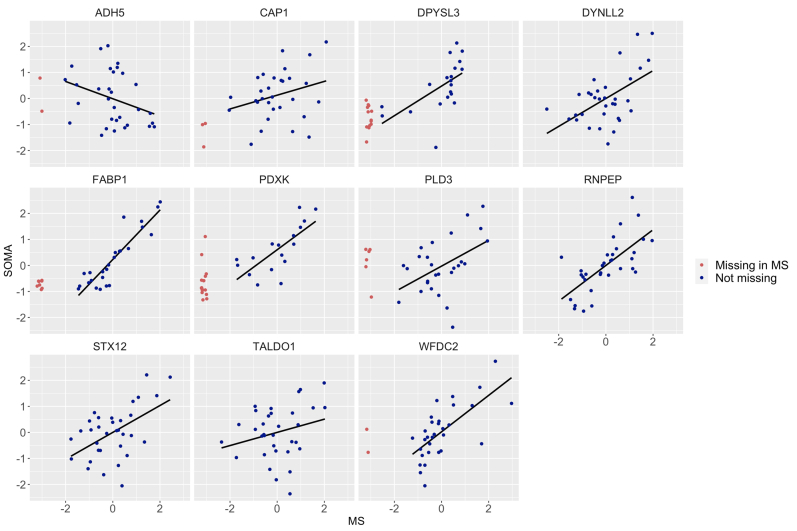
**Scatter plots of the 11 proteins** identified by **one aptamer in the SOMAscan assay.** On the x-axis: log2-transformed and standardized mass spectrometry (MS) data. On the y-axis: log2-transformed and standardized SOMAscan assay data. Data from all three groups are included. Samples with missing data in MS are visualized in *red* to show their value in the SOMAscan assay data set.

**Supplemental Figure S6:**
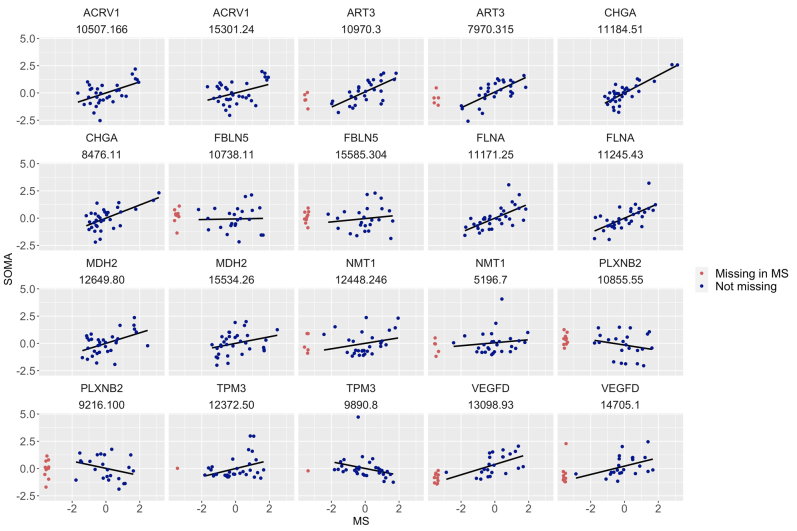
**Scatter plots of the 10 proteins** identified by **two aptamers in the SOMAscan assay.** On the x-axis: log2-transformed and standardized mass spectrometry (MS) data. On the y-axis: log2-transformed and standardized SOMAscan assay data. First, the gene name is given and below, the aptamer seq-id is given (for example gene ACRV1 with aptamers 10507.166 and 15301.24). Data from all three groups are included. Samples with missing data in MS are visualized in *red* to show their value in the SOMAscan assay data set.

**Supplemental Figure S7:**
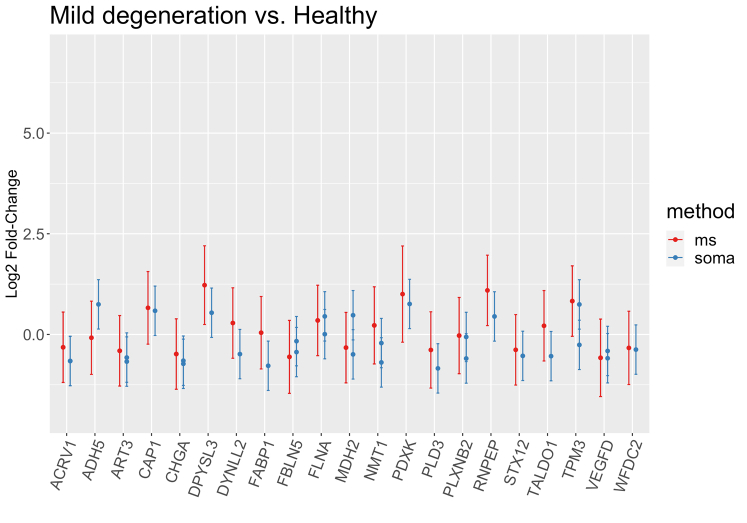
**The log2 fold change for proteins for both methods of mass spectrometry (MS) proteomics (*red*) and SOMAscan assay proteomics (*blue*).** The text on the x-axis is the Entrez Gene. Two data points for the SOMAscan means that the corresponding protein was detected by two targeted aptamers. The comparison is for mild degeneration to healthy. Bars show the 95% CIs.

**Supplemental Figure S8:**
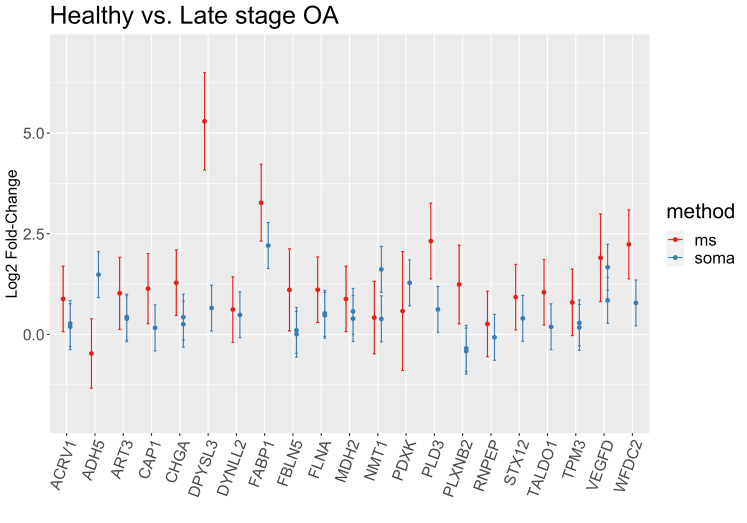
**The log2 fold change for proteins for both methods of mass spectrometry (MS) proteomics (*red*) and SOMAscan assay proteomics (*blue*).** The text on the x-axis is the Entrez Gene. Two data points for the SOMAscan means that the corresponding protein was detected by two targeted aptamers. The comparison is for healthy to late stage OA. Bars show the 95% CIs

**Supplemental Figure S9:**
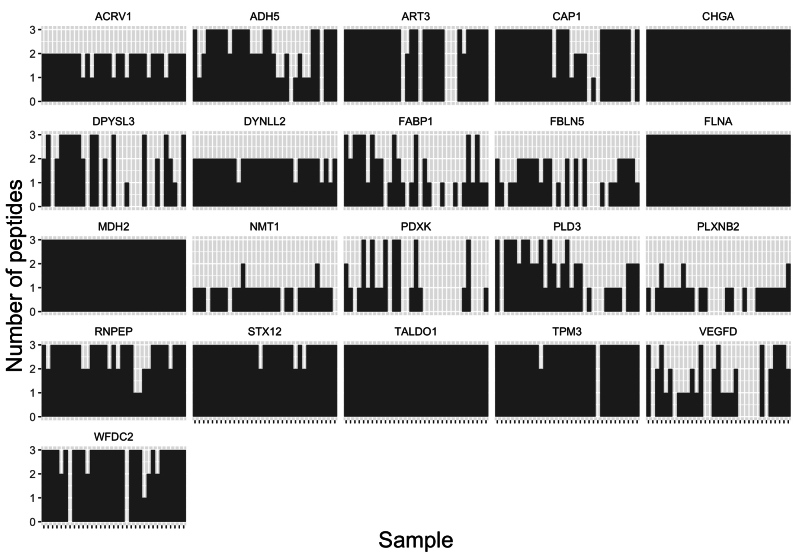
**Quantification of proteins were done within Spectronaut 18 of the 3 most abundant peptides.** However, there is not always 3 peptides within each sample eligible for quantification. These plots show how many peptides (y-axis) that were used for quantification in each sample (x-axis). Data from all three groups are included.

We focused on 21 proteins that were included in the GGMs for validation purposes (Supplemental Datas S7 and S8). Scatter plots between MS and SOMAscan assay data show that there is a positive correlation between the protein abundance measured with the two methods for most proteins (Supplemental Figs. S5 and S6). The comparison of log2 fold changes and 95%CIs (Supplemental Datas S9 and S10) between the methods show similar results for the mild degeneration group when compared to healthy controls (Supplemental Fig. S7), whereas the results for the comparison between healthy controls and late-stage OA group show dissimilarities for a few proteins (Supplemental Fig. S8). The negative correlation for some of the proteins can potentially be explained by that the quantification, in each sample, was based on only one or a few peptides (Supplemental Fig. S9). Worth to highlight is that the signal for all transitions for plexin-B2 fragments was low when compared to transitions in the same m/z-region (data not shown) and was the protein with lowest coverage among the 21 proteins (Supplemental Data S7). Further, alcohol dehydogenase class-3 had two missed cleavages (data not shown) for one of the peptides which were used for quantification in all samples with non-missing values. Both these proteins show a negative correlation.

### SLCO5A1: Antibody Validation by Western Blotting

We identified SLCO5A1 in the sample with the highest abundance according to the SOMAscan assay. After the removal of the most abundant proteins, we were able to achieve distinct bands close to the predicted molecular weight of 92 kDa (Supplemental Fig. S10).

## Discussion

In this exploratory study, we investigated the conditional dependencies of SF proteins in healthy human knees and knees with visual signs of mild degeneration using a novel GGMs approach. To our knowledge, this is the first study that in a data-driven manner explores protein networks from SF in a group that may represent early-stage OA (37).

We have identified several proteins that have previously been reported to be involved in OA. Recently, a literature data mining approach was utilized to create a list of proteins involved in OA disease (38). In comparison to this list, we identified 1264 of the 1676 proteins that the authors identified as present in tissue and biofluid of the knee. Furthermore, we have previously collected SF data from a similar cohort which were subjected to nano liquid chromatography-MS/MS (12). For the late-stage OA group in our previous study, the proteins decorin, PPBP and MYOC (Entrez Gene) had the highest absolute fold changes. These proteins are among the ones with the highest absolute fold change for the late-stage OA group in this study as well (Supplemental Data S2). Among differentially expressed proteins for the mild degeneration group, we found for example PADI4, which has previously been studied regarding rheumatoid arthritis (RA) (39, 40, 41), but has also been detected in synovial membrane in regard to OA (38).

In this study, however, we wanted to expand the analysis of the proteome by looking at the protein as a whole network and not only perform differential expression analysis. We conducted the data exploration without incorporating *a priori* information and found a higher degree of network complexity in healthy knees. The lost interactions observed in mild degeneration might signify disruptions in the normal interplay among these proteins, possibly contributing to a disease state. Some of the proteins with unique edges in the healthy group, ELMO1, NMT1 and PTPN11 (Table 1), have previously been reported to be associated with either OA or inflammatory arthritis. A study found reduced joint inflammation and alleviated disease severity through ELMO1's regulation of neutrophil recruitment to inflamed joints in mice (42). NMT1 has been reported to have tissue-protective functions, while loss of NMT1 caused synovial tissue inflammation (43). The protein PTPN11 mediates cellular responses to hormones and cytokines (44) and was overexpressed in fibroblast-like synoviocytes in RA patients compared to OA patients (45). Overall, these previous findings align with the trend of downregulation in the current study. In the network with unique edges in the healthy group, the protein with the highest betweenness score, SLCO5A1, has, to our knowledge, not previously been associated with OA. SLCO5A1 is a member of an organic anion-transporting polypeptide (OATP) family (46), a group of proteins that mediate transport across the cell membrane. Altered transportation of certain substances, such as chemokines and metabolites, might affect inflammatory responses, cell signaling, or metabolism within the joint, potentially impacting the disease processes in OA (47, 48, 49, 50). We identified SLCO5A1 in the sample with the highest abundance (according to SOMAscan assay data) using Western blot. However, further research is needed to investigate the role of OATPs in OA.

**Table 1:** Proteins with unique edges in healthy group’s network

Community	Entrez Gene	Classification	Degree centrality	Betweenness centrality	Log2 fold change	95% CI
B	ELMO1	scaffold/adaptor protein	12	431	−0.43	(−0.85, −0.02)
B	RNPEP	*NA*	29	426	0.44	(−0.09, 0.97)
B	NMT1	transferase	15	279	−0.55	(−1.10, 0.00)
C	PTPN11	protein phosphatase	27	341	0.44	(−0.04, 0.92)
C	LRRN1	transmembrane signal receptor	21	277	−0.43	(−0.96, 0.10)
D	SLCO5A1	transporter	32	535	−0.44	(−0.99, 0.10)
D	ADH5	dehydrogenase	30	390	0.62	(0.00, 1.26)
E	PTS	*NA*	31	454	0.49	(0.01, 0.96)
E	CAP1	actin or actin-binding cytoskeletal protein	34	365	0.48	(0.04, 0.93)
E	POLR3F	DNA metabolism protein	27	249	−0.46	(−0.89, −0.02)

The 10 proteins with highest betweenness scores are displayed. This table represents these proteins’ community, Entrez Gene, Classification (where applicable), centrality scores (degree and betweenness), Log2 fold change, and 95% confidence interval (95% CI). A positive Log2 fold-change means that the protein is upregulated in the mild degeneration group as compared to healthy controls.

Previous studies on SF proteomics specifically have used different methods and platforms to differentially quantify the proteome of OA patients *versus* healthy controls, such as 2D gel electrophoresis (51), liquid chromatography-MS (52) and immunoassays (12). Most of these studies focused on late-stage OA patients, which may not necessarily reflect the early molecular changes that initiate OA pathogenesis. While the proteins identified by the SOMAscan assay cover a substantial array of proteins previously reported to be associated with OA, few of the proteins with unique edges in the mild degeneration group (Table 2) have been mentioned in previous studies. The novel findings in this study may be due to the sensitivity of the SOMAscan assay, which has several advantages over more conventional proteomics approaches, including wide dynamic range, low variability, and high reproducibility (53). The novelty of our results may also be attributable to our unique GGMs network approach and study population of early-stage OA individuals. However, SOCS3, a negative regulator of cytokines, has previously been studied regarding OA (54, 55, 56). One study observed an increase in expression of SOCS3 in chondrocytes obtained from joint replacement patients (54), while we could not confirm this (late-stage OA compared to healthy controls, log2 fold change 0.084, 95% CI [−0.43, 0.60], Supplemental Data S2). Furthermore, the protein expression is decreased in the mild degeneration group (Table 2), suggesting a possible shift in protein expression during disease progression. Interestingly, SOCS3 has been suggested to potentially both protect osteoarthritic joints by its anti-inflammatory effect as well as delay tissue repair by reduced chondrocyte growth (56). One of the other proteins with a high betweenness centrality score was the APOBEC1 complementation factor (A1CF) (Table 2). A1CF has been described in the context of gout, a form of inflammatory arthritis, and hyperuricemia (57). Further, there is evidence of the involvement of Histone H1.10 (H1-10) in RA, suggesting that citrullination of H1-10 could be a specific marker for RA (58). To our knowledge, the other proteins we identified as potential bottleneck proteins in the mild degeneration group (HIBCH, DHX8 and PHF3), based on betweenness centrality, have not previously been discussed in relation to OA and require further validation. These proteins have been described to be involved in valine catabolism (HIBCH) (59), splicing events (DHX8) (60) and regulation of transcription and RNA processing (PHF3) (61). We expected ECM proteins to be more prominent in our analysis considering the degenerative nature of knee tissues in OA. However, the group we analyzed represents the potential early stage of the disease, with only mild degeneration of cartilage and/or meniscus.

To strengthen our results, we also collected MS data. Overall, between group differences based on SOMAscan and MS data were consistent (Supplemental Fig. S6), and for most proteins, there was a positive correlation between values from the two methods (Supplemental Figs. S4 and S5). One of the advantages of SOMAscan assay over MS proteomics is the ability to detect low-abundant proteins. However, aptamers in SOMAscan are designed to bind the native form of proteins (18) which consequently reveals one limitation of the method. If epitope availability is affected by the disease state, through for example post-translational modifications (PTMs), this cannot be captured with the method. The dysregulation of several types of PTMs have been associated with OA (62, 63, 64, 65), which could potentially explain the lack of agreement between the methods for some of the proteins. This is, however, highly speculative, and the investigation of PTMs is out of scope for this study. Neither SLCO5A1 or one of the proteins with the highest betweenness in the mild degeneration network (HIBCH, DHX8, A1CF and PHF3) was detected with MS. However, HIBCH and DHX8 have previously been confirmed by MS after enrichment with aptamers from the SOMAscan assay (66).

One limitation of the study is the use of both pre- and post-mortem samples because alterations of the proteome are still occurring after death (67). It has been shown that degradation is specific to the organ, but sampling within 24 h is probably adequate for most organs (68). Our main goal was, however, to analyze network models from two groups, healthy and mild degeneration, where the tissue was obtained post-mortem for both groups, which hopefully made the impact of protein alteration due to death less of an issue. Another limitation of this study is the relatively low sample size. A well-known consequence of high throughput data and low sample size is model overfitting. Therefore, validation of model construction on other datasets should be conducted. To address this issue, however, we used a regularization approach to estimate the GGMs, which can reduce the number of false positives and improve the model fit (19). We also used a stability selection procedure to further select the most reliable edges in the GGMs. However, due to the high dimensionality of the data (7088 aptamers) and the low number of samples (25), we had to reduce the number of aptamers to be included in the GGMs. We chose proteins based on their absolute log2 fold change values from the differential expression analysis, assuming these would be the most “interesting” proteins. This may introduce some bias and miss important proteins that are not differentially expressed but that may be differentially connected.

In the final models, after applying stability selection with 1000 subsets, only 103 aptamers were retained. One interpretation of this reduction is that a vast majority of the aptamers are *not* conditionally dependent. Another interpretation is that the stability selection is too stringent. Selection of the regularization parameters for GMMs is challenging and no gold standard approach exists so far (29). We used BIC to select the optimal regularization parameters for the GGMs, while also evaluating the sparseness of the models. We aimed for networks that are complex enough to be interesting but sparse enough to be interpretable (29). We considered this trade-off suitable given the exploratory and hypothesis-generating nature of this study.

## Conclusion

We have performed proteomic analysis of SF from healthy and mildly degenerated knee joints, using the SOMAscan assay and GGMs. This study contributes to the field of OA biomarker discovery, by providing a more complete and accurate mapping of the synovial fluid proteome in a group representing early OA. We identified several proteins and communities that are differentially abundant or connected between the two groups, suggesting potentially important mechanisms for early OA pathogenesis. Our study provides new insights into the complex and dynamic nature of protein interactions in OA pathogenesis and may pave the way for better understanding and treatment of this disease.

## Data Availability

The data that support the findings of this study are available from the corresponding author upon reasonable request. The SOMAscan assay data have been deposited in the Zenodo data repository with the dataset identifier https://doi.org/10.5281/zenodo.8247424. The R code used in the analysis and visualization of this data is available at https://github.com/martinry/sf-ggm. The mass spectrometry proteomics data have been deposited to the ProteomeXchange Consortium *via* the PRIDE (69) partner repository with the dataset identifier PXD051182. The raw data contains six additional biological samples, which were not analyzed by the SOMAscan assay, as well as seven technical replicates. All samples were used in the Spectronaut search.

## Supplemental data

This article contains supplemental data.

## Conflict of interest

The authors declare no competing interests.

## Supplementary data

Supplemental Figure S1 **Representative images of fem****oral****articular cartilage****from the groups healthy (*left*; male, 49 years old) and mild degeneration (right; male, 73 years old).**

Supplemental Figure S3 **Protein-protein interaction networks derived from Gaussian Graphical Models (GGMs) for two study groups: healthy controls (*Panel A*) and mild degeneration (*Panel B*).** Each node in the network signifies a protein, with edges representing conditional dependencies or interactions between two proteins. Frames of nodes are colored *blue* and *red* to indicate upregulated and downregulated proteins (mild degeneration in comparison to healthy controls), respectively. The *grey* edges represent paths that are shared between the two groups, whereas the *green* and *orange* edges are unique for the individual groups. Proteins are displayed in the same position in both images. The Fruchterman-Reingold algorithm was used for layout and the same protein may have different positions in the two networks. The absence of a node indicates that the corresponding protein had no detected interactions in either condition.

Supplemental Figure S4 **Protein-protein interaction networks derived from Gaussian Graphical models (GGMs) for two study groups: healthy controls (*Panel A*) and mild degeneration (*Panel B*).** Only nodes with unique edges in the respective group were included. Each node in the network signifies a protein, with edges representing conditional dependencies or interactions between two proteins. Frames of nodes are colored *blue* and *red* to indicate upregulated and downregulated proteins (mild degeneration in comparison to healthy controls), respectively. Community detection was performed using the cluster_louvain function in R. Communities with more than 2 members are colored and labelled A-K. *Black* edges indicate interactions within a community, and red edges indicate interactions between communities. The Fruchterman-Reingold algorithm was used for layout and the same protein may have different positions in the two networks. The absence of a node indicates that the corresponding protein had no detected interactions in either condition.

Supplemental Figure S10 **Western blot analysis of 10 μL, 6 μL and 4 μL SF****from****the sample with highest abundance of SLCO5A1 according to the SOMAscan assay.** Whole gel (*left*) and lanes with samples only (*right*). The bands are close to the predicted molecular weight of 92 kDa. The band for a SF volume of 4 μL is almost not visible, suggesting that the available epitopes in the sample are low.
