# Supplementary figures and images for: Exploring the Early Molecular Pathogenesis of Osteoarthritis Using Differential Network Analysis of Human Synovial Fluid
Source: Mol Cell Proteomics. 2024 May 14;23(6):100785. doi: 10.1016/j.mcpro.2024.100785 (PMC11252953; doi:10.1016/j.mcpro.2024.100785)

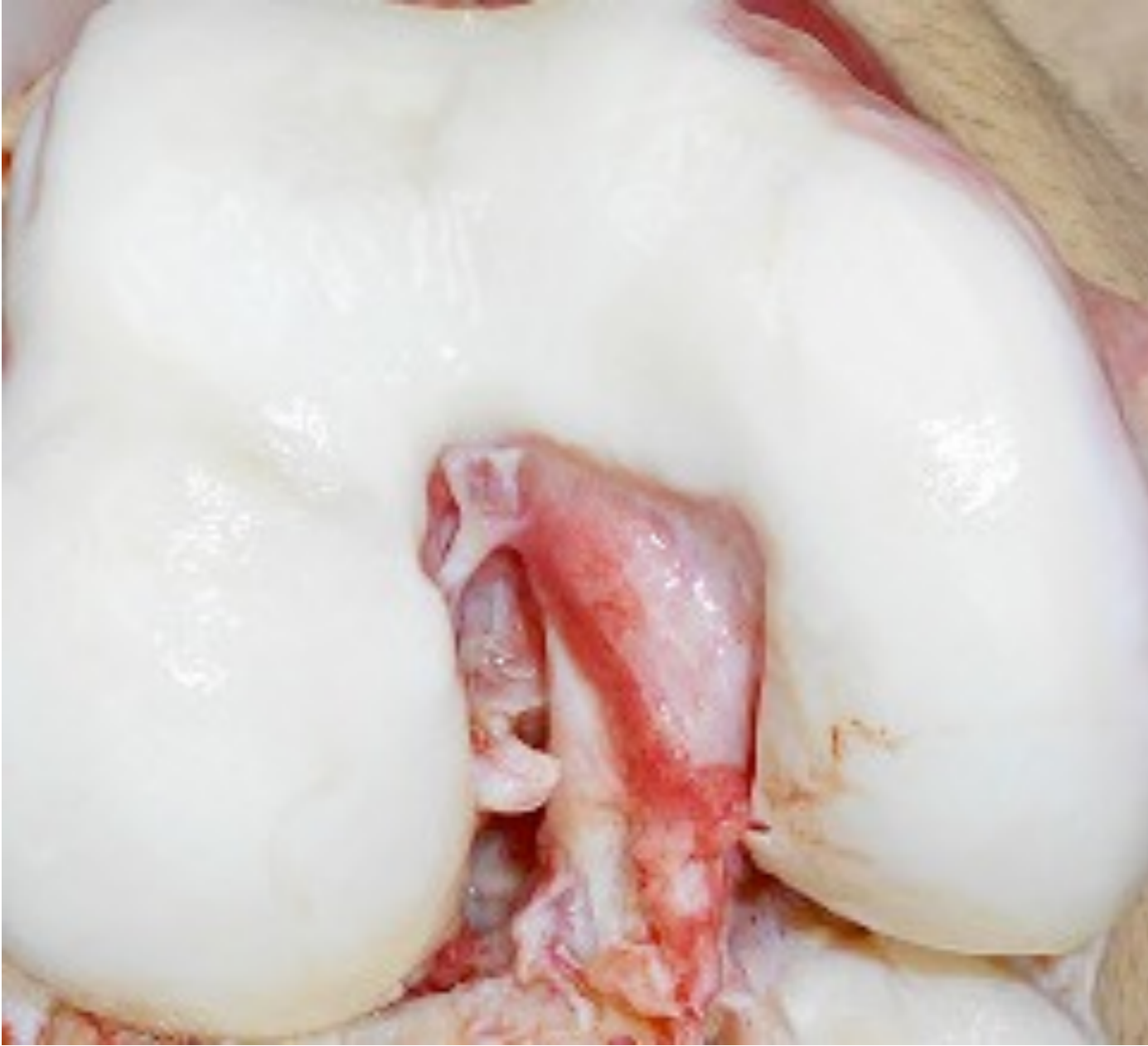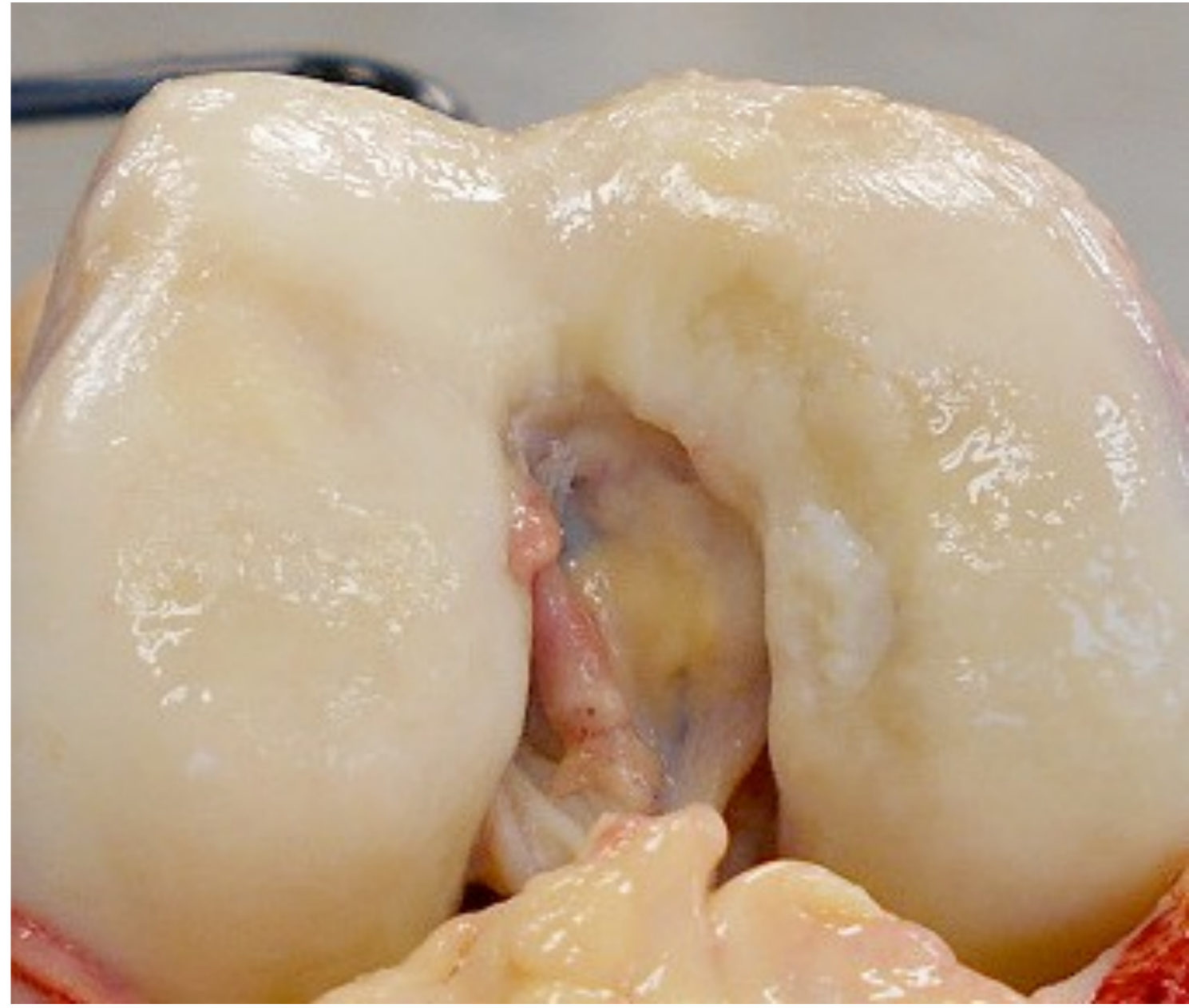

Supplement: Supplemental Figure S1 — Representative images of femoralarticular cartilagefrom the groups healthy (left; male, 49 years old) and mild degeneration (right; male, 73 years old). [file mmc1.pdf]

**HEALTHY**

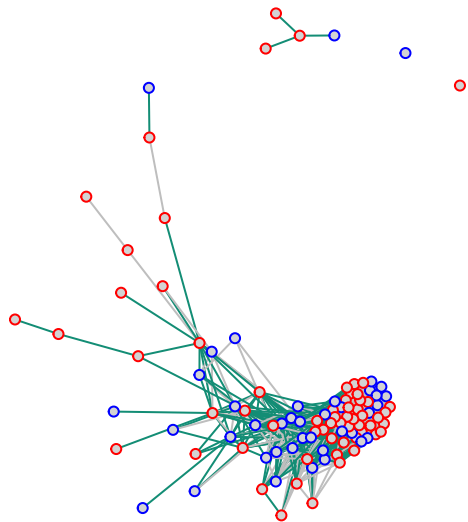

**MILD DEGENERATION**

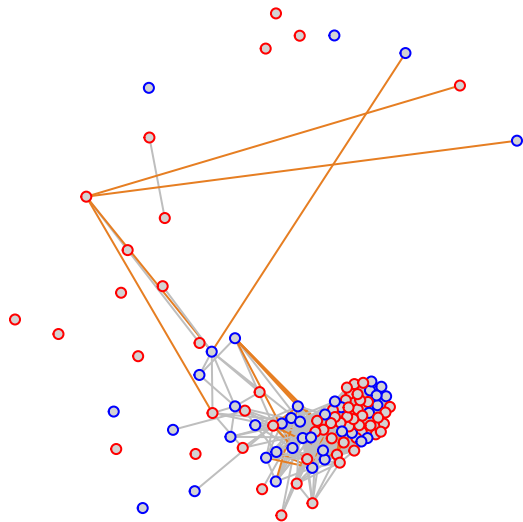

Supplement: Supplemental Figure S3 — Protein-protein interaction networks derived from Gaussian Graphical Models (GGMs) for two study groups: healthy controls (Panel A) and mild degeneration (Panel B). Each node in the network signifies a protein, with edges representing conditional dependencies or interactions between two proteins. Frames of nodes are colored blue and red to indicate upregulated and downregulated proteins (mild degeneration in comparison to healthy controls), respectively. The grey edges represent paths that are shared between the two groups, whereas the green and orange edges are unique for the individual groups. Proteins are displayed in the same position in both images. The Fruchterman-Reingold algorithm was used for layout and the same protein may have different positions in the two networks. The absence of a node indicates that the corresponding protein had no detected interactions in either condition. [file mmc2.pdf]

## HEALTHY

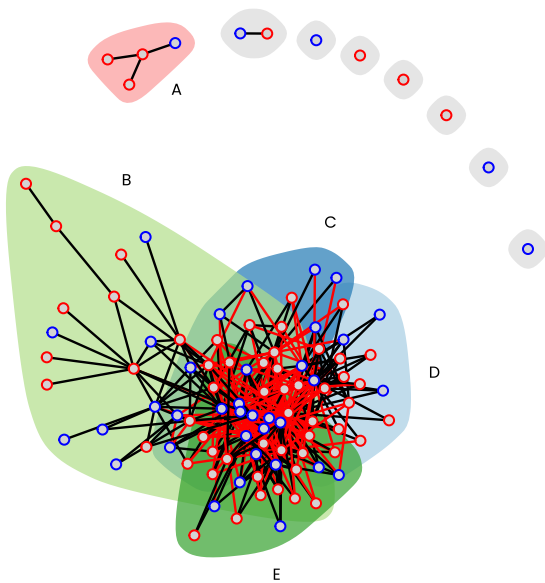

## MILD DEGENERATION

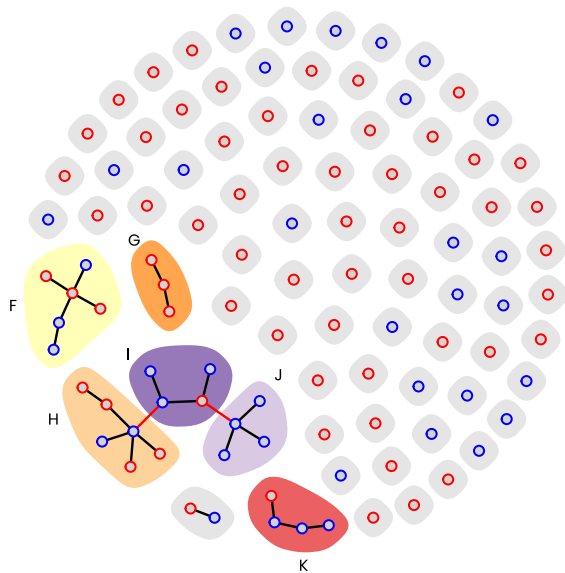

Supplement: Supplemental Figure S4 — Protein-protein interaction networks derived from Gaussian Graphical models (GGMs) for two study groups: healthy controls (Panel A) and mild degeneration (Panel B). Only nodes with unique edges in the respective group were included. Each node in the network signifies a protein, with edges representing conditional dependencies or interactions between two proteins. Frames of nodes are colored blue and red to indicate upregulated and downregulated proteins (mild degeneration in comparison to healthy controls), respectively. Community detection was performed using the cluster_louvain function in R. Communities with more than 2 members are colored and labelled A-K. Black edges indicate interactions within a community, and red edges indicate interactions between communities. The Fruchterman-Reingold algorithm was used for layout and the same protein may have different positions in the two networks. The absence of a node indicates that the corresponding protein had no detected interactions in either condition. [file mmc3.pdf]

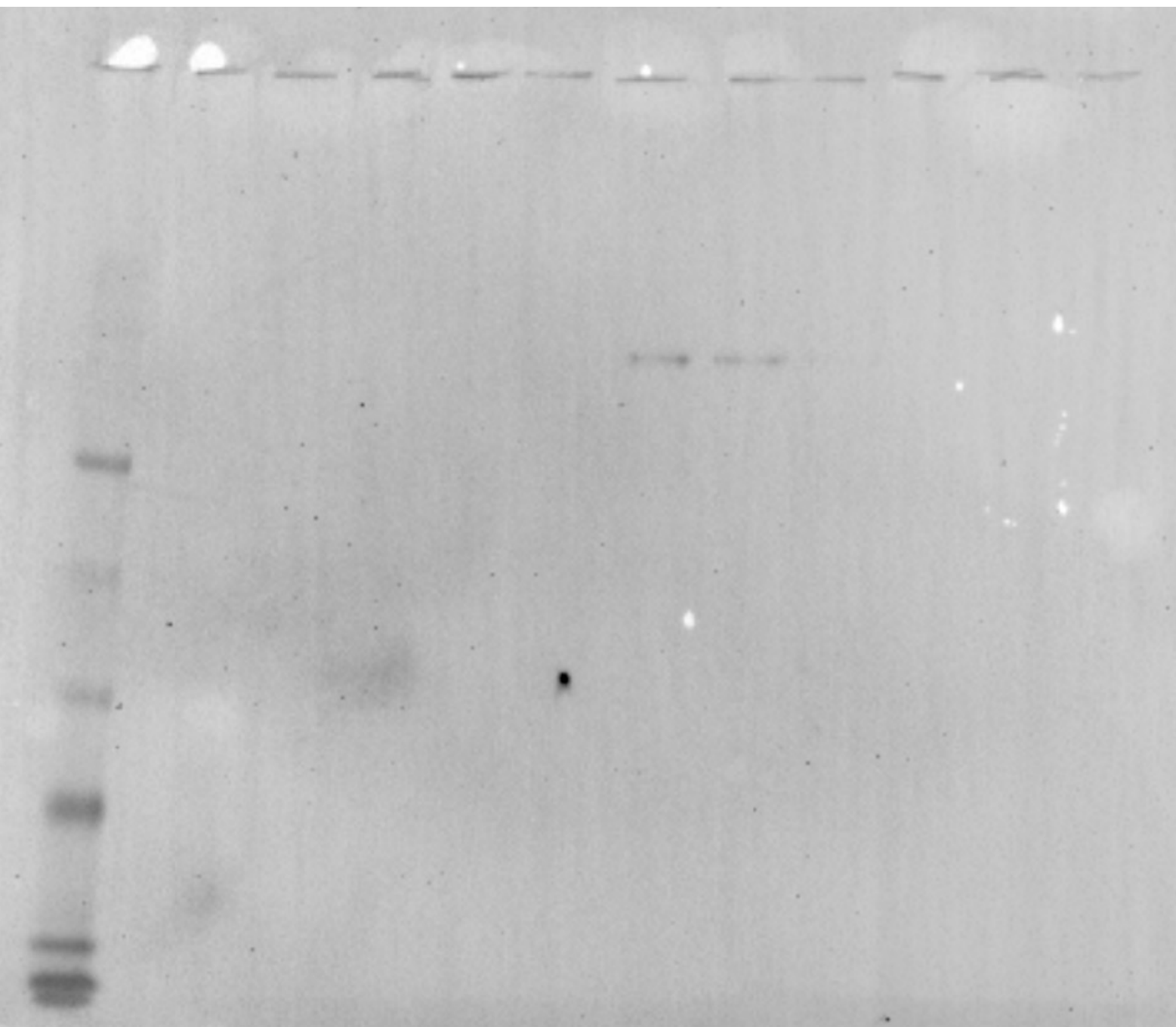

191 —

97 —

64 —

51 —

39 —

28 —

19 —

14 —

10  $\mu$ L SF

6  $\mu$ L SF

4  $\mu$ L SF

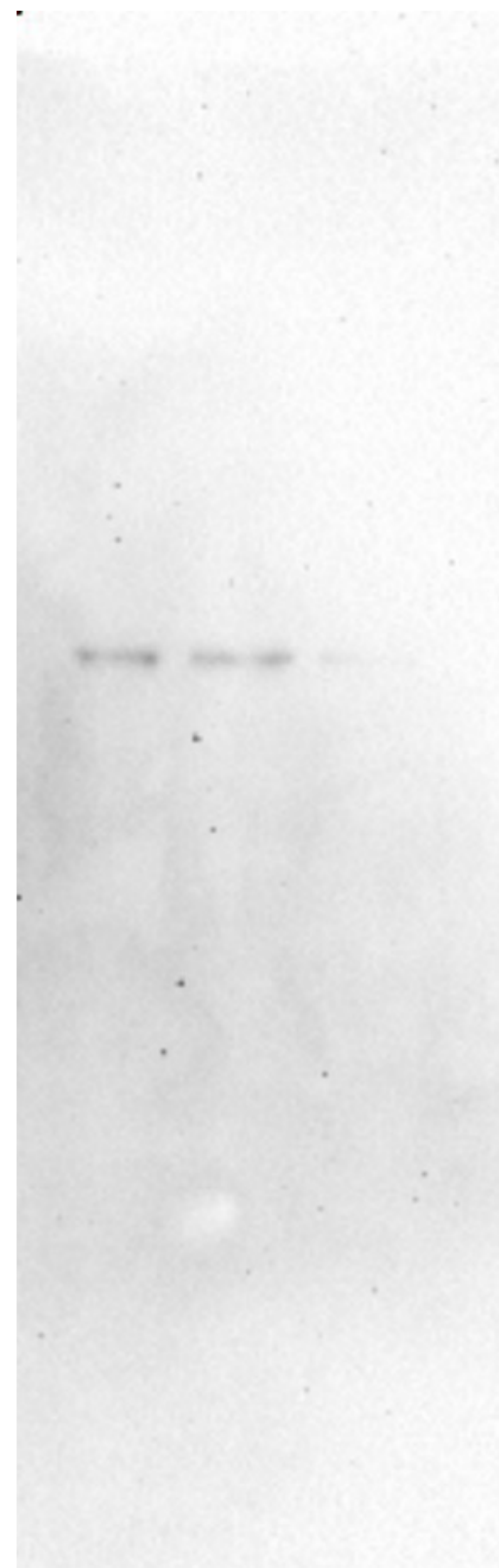

Supplement: Supplemental Figure S10 — Western blot analysis of 10 μL, 6 μL and 4 μL SFfromthe sample with highest abundance of SLCO5A1 according to the SOMAscan assay. Whole gel (left) and lanes with samples only (right). The bands are close to the predicted molecular weight of 92 kDa. The band for a SF volume of 4 μL is almost not visible, suggesting that the available epitopes in the sample are low. [file mmc4.pdf]
